# Microsatellite Instability and Altered Expressions of *MLH1* and *MSH2* in Gastric Cancer

**DOI:** 10.31557/APJCP.2019.20.2.509

**Published:** 2019

**Authors:** Nor Hasyimah Haron, Ezanee Azlina Mohamad Hanif, Mohd Rizal Abdul Manaf, Jasmi Ali Yaakub, Roslan Harun, Ramelah Mohamed, Isa Mohamed Rose

**Affiliations:** 1 *Department of Pathology,*; 2 *UKM Medical Molecular Biology Institute (UMBI), *; 3 *Department of Community Health, University Kebangsaan Malaysia Medical Centre, Jalan Yaacob Latif, 56000 Cheras, Kuala Lumpur,*; 4 *General Surgery, KPJ Ampang Puteri Specialist Hospital, No. 1 Jalan Mamanda 9, Taman Dato Ahmad Razali, 68000 Ampang, Selangor Darul Ehsan, Malaysia.*

**Keywords:** Gastric cancer, microsatellite instability, immunohistochemistry, microsatellite analysis

## Abstract

**Introduction:**

Microsatellite instability (MSI) is a hallmark of defective DNA mismatch repair (MMR) of genes especially *MLH1* and *MSH2*. It is frequently involved in the carcinogenesis of various tumours including gastric cancer (GC). However, MSI in GCs have not been reported in Malaysia before. Objective: This study was conducted to determine the microsatellite instability (MSI) status in gastric cancer by microsatellite analysis, sequencing, its association with *MLH1* and *MSH2* protein expression and *H.pylori* infection by immunohistochemistry.

**Method:**

A total of 60 gastric cancer cases were retrieved. DNA was extracted from paired normal and tumour tissues while *MLH1* and *MSH2 *protein expression as well as H. pylori status were determined by IHC staining. For microsatellite analysis, polymerase chain reaction (PCR) was performed for paired tissue samples using a panel of five microsatellite markers. MSI-positive results were subjected for DNA sequencing to assess mutations in the *MLH1* and *MSH2* genes.

**Results:**

Microsatellite analysis identified ten MSI positive cases (16.7%), out of which only six cases (10.3%) showed absence of *MLH1* (n=3) or *MSH2* (n=3) protein expression by IHC. The most frequent microsatellite marker in MSI positive cases was BAT26 (90%). Nine of ten MSI positive cases were intestinal type with one diffuse and all were located distally. *H. pylori* infection was detected in 13 of 60 cases (21.7%) including in three MSI positive cases. All these results however were not statistically significant. Our sequencing data displayed novel mutations. However these data were not statistically correlated with expression levels of *MLH1* and *MSH2* proteins by IHC. This may be due to small sample size to detect small or moderately sized effects. **Conclusion:** The frequency of MSI in this study was comparable with published results. Determination of affected MMR genes by more than two antibodies may increase the sensitivity of IHC to that of MSI analysis.

## Introduction

Previous studies evidently showed 4 distinct subtypes of GCs; one with high infection of Epstein-Barr virus, microsatellite insability that leads to high hypermethylation status in *MLH1* gene, genomic instability in diffused GC histology, and chromosomal instability with vast numbers of somatic copy number aberrations mostly in intestinal histological types of GCs (Wong et al., 2015). Microsatellite instability (MSI) accounted for 13-44% of GCs depending on the number of cases and remains as the hallmark of GCs (Moskaluk and Rumpel, 1998; Wirtz et al., 1998). A panel of five microsatellite markers (NCI panel) were recommended by National Cancer Institute in 1997 to provide some uniformity in defining MSI positive tumours in colorectal cancer (Boland et al., 1998). However, the efficiencies of these markers associated with extracolonic cancers including GC remains uncertain and need to be evaluated.

Microsatellite instability is a resultant of defective DNA mismatch-repair (MMR) genes including* hMSH2*, *hMLH1*, *PMS1*, *PMS2*, *hMSH6* and* hMSH3*. Germline mutations of hMLH1 and hMSH2 are among the most frequently found in hereditary nonpolyposis colon carcinoma (HNPCC) or Lynch Syndrome (Han et al., 1995). Microsatellite instability refers to changes in at least a single to two or more nucleotide repeat segments known as microsatellites that are distributed throughout the genome (Ionov et al., 1993). 

GC with presence of MSI is associated with certain clinicopathological characteristics such as older age of presentation, distal tumour location, intestinal type by Laurèn classification, expanding type by Ming classification, no lymph metastasis, early disease staging, and better overall prognosis (Lee et al., 2002; Falchetti et al., 2008; Kim et al., 2011). Furthermore, a positive correlation between the presence of *H. pylori* seropositivity and MSI GCs have been observed (Simpson et al., 2000; Scartozzi et al., 2004). Active *H. pylori* infection is present more frequently in individuals carrying MSI GCs, suggesting that *H. pylori* may affect MMR system during the stepwise progression of gastric carcinogenesis.

In Malaysia, 82% GC cases were detected in the late stage (stage IV) and about 16% were detected in early stage (Stage I and II) (Kandasami et al., 2003). More recent data from the Malaysia Cancer Statistics – Data and Figure 2007 report showed almost similar figures of 73.4% of cases detected in Stages III and IV. This situation has drawn attention to the late detection of cancer as an important negative factor in the Malaysian setting. Additionally, limited research has been conducted in exploring the background of GC genetics in Malaysia. The findings of this study could add on to the existing knowledge linked to GCs. This retrospective study intended to determine the frequency of MSI in 60 cases of resected GCs using immunohistochemical expression of MLH1 and MSH2 protein and comparing it with PCR-based MSI analysis using a panel of five MSI markers. MSI positive cases were also screened for mutations to see whether there is any strong correlation with MSI status, *H. pylori* status, *MLH1* and *MSH2* expressions in GCs. 

## Materials and Methods

*Patients and Tissue Samples*

Matched pairs of formalin-fixed, paraffin embedded normal and tumour tissue samples were obtained from 60 cases of resected gastric carcinoma from the UKM Medical Centre (UKMMC) following approval from the Ethics Committee of UKMMC. Normal samples were obtained from the matched adjacent GC samples on different FFPE blocks. None of the cases had a sporadic or familial history suggestive of HNPCC syndrome. The clinical information was obtained from pathology reports. A pathologist was referred for the purpose of histopathology diagnoses verification. In total, 39 males and 21 females consisted of 44 Chinese, 14 Malays and 2 Indians were included. The patients were between 28 to 83 years (mean age 64 years). There were 38 intestinal and 22 diffuse cancers confirmed by the Laurén classification. Hematoxylin and eosin–stained (HNE) sections of paraffin blocks were examined, and representative samples were selected for further analysis. 5µm of representative samples were sectioned and laid on coated poly-L-lysine (Sigma Aldrich, St. Louise, USA) slides for immunohistochemical analysis. Approximately, 2 – 10 µm sections from normal and tumour blocks were subjected for DNA isolation using ‘QIAamp DNA Mini Kit’ (QIAGEN, GmbH, Germany). 

*Publically available data*

Survival rate of MLH1 and MSH2 expression levels by Kaplan Meier Plotter Database.

Survival curves were generated from the Kaplan Meier Plotter (Gastric Cancer) database based on the expression levels of the MLH1 and MSH2 from publically available data of 1,065 GC patients. Graphs were generated by taking into account all types of treatments (surgery only, 5-fluorouracil adjuvant and other adjuvant chemo), HER2 status, all stages (TNM), Lauren classification (Intestinal, diffuse and mixed), Differentiation (well, moderate and poor), and gender as selection criteria (Szász et al., 2016).

*Immunohistochemical Staining of MMR proteins (hMLH1, hMSH2) and H. pylori*

Mouse monoclonal antibodies against hMLH1 (BD Pharmingen, USA) and hMSH2 (Calbiochem, USA) with a dilution factor at 1:50 and 1:150 were used for immunostaining. The immunostaining was performed with Envision Horseradish peroxidase kit (DakoCytomation, Denmark) for hMLH1 and RealTM EnVisionTM Detection Kit (Lab Vision Corporation, Canada, USA) for hMSH2. Diaminobenzidine (DakoCytomation, Denmark) was used as a chromogen. Sections were lightly counterstained with hematoxylin (JT Baker, Holland) for 30 seconds. Absence of MSI was determined by a brownish nucleus staining with DAB. Loss of hMSH2 or hMLH1 expression in cancer tissues was demonstrated by undetectable nuclear staining of neoplastic cells indicated the genes were affected.

*Analysis of MSI*

MSI was detected using the National Cancer Institute (NCI) panel of 5 microsatellite markers; BAT25, BAT26, D2S123, D5S346 and D17S250 in normal and tumour tissues. Primer sequences were as described previously (Loukola et al., 2001) and each sense primers were end-labeled with fluorescent marker, Hex. This process was performed by PCR in a total volume of 25 µl consisted of 150-300 ng DNA, 12.5 µl ImmoMixTM (Bioline, UK), and 1 x sense and antisense primers each. PCR amplification was conducted on MyCycler thermocycler (Biorad, USA). Samples were denatured at 95°C for 7 minutes, followed by 40 cycles of denaturation (94°C for 1 minute), annealing (55°C for 1 minute), extension (72°C for 1 minute) and ultimately by final extension at 72°C for 7 minutes and were kept hold at 4°C. Then, 1µl of fluorescently labeled PCR products was mixed with 12µl deionized formamide and 1µl GeneScan TAMRA 500 Size Standard (Applied Biosystem, Carlsbad, CA, USA). The mixture was denatured at 94°C for 5 minutes and hold at 4°C before loaded to the ABI PRISM 3130XL Genetic Analyzer (Applied Biosystem). 

The data was analysed using Gene Mapper software (Applied Biosystems), which automatically determined the actual size of PCR products and the amount of fluorescent signal from electropherograph outputs. MSI was predicted by the presence of novel peaks in tumour tissue compared to control. The second indicator for MSI was the difference in microsatellite length in these samples. Tumours exhibited MSI with two or more markers were defined as MSI positive.

*Sequencing*

Samples that displayed positive MSI were subjected for direct sequencing. The DNA samples were set up for sequencing using BigDye Terminator v1.1 Cycle Sequencing kit (Applied Biosystems) consisted of 2µl BigDye buffer, 1.6µl Sequencing primer, 0.2µl BigDye Enzyme and 100ng DNA. Sequencing cycle condition was set up accordingly on Genetic Analyzer (Applied Biosystems). 

*Statistical Analysis*

The sensitivity and specificity of MLH1 or MSH2 immunostaining in identifying MSI positive tumours were calculated. Sensitivity was defined as the absence of MLH1 or MSH2 expressions by IHC in MSI positive tumours, while specificity was defined as intact expressions of MLH1 and MSH2 by IHC in MSI negative tumours. Tests for differences between MSI positive and MSI negative groups were performed using either the chi-square test or the Fisher’s exact test. P value ≤ 0.05 were considered to be statistically significant.

## Results

*MLH1 and MSH2 expression levels are associated with poor survival in gastric cancers*

Survival curves of MLH1 and MSH2 were generated from the KM-plotter database, showed significant poor overall survival in high MLH1 and MSH2 expressions. This indicated that these markers might represent as a good prognostic value in GCs.

*Microsatellite Instability*

Ten cases (10/60, 16.7%) showed MSI positive by microsatellite analysis. Out of which only six cases (10.3%) showed absence of protein expression, three by loss of MLH1 (Figure 2A) and three by MSH2 (Figure 2B) via immunostaining. The rest 50 cases (83.3%) were considered MSI negative. The sensitivity and specificity rates for IHC testing assessed against MSI positive results were 60% (6 of 10) and 100% (50 of 50) respectively. The most frequently positive of the panel markers was BAT26 (90%) whilst the rest D17S250, BAT25, D5S346 and D2S123 being 60%, 50%, 30% and 20% respectively. Figure 3 showed an example of electropherogram reading from Genotyper Analysis Software (Applied Biosystems) for a case with MSI positive. This case showed instability for BAT25 and BAT26 markers. Table 1 showed comparison of microsatellite analysis with MSH2 and/or MLH1 immunohistochemical staining. There were four MSI positive cases with intact MLH1 and MSH2 protein expressions. Table 2 displayed clinicopathological characteristics of ten MSI positive cases, involving eight Chinese (six males and two females) and two Malay females. Of the ten MSI positive cases, nine were of intestinal type and one diffuse. *H. pylori* infection was detected in normal gastric mucosa in 13 of 60 cases (21.7%) including three MSI positive cases by IHC (Figure 4). However, all these results were statistically insignificant (Table 3).

**Table 1:** Comparison of Microsatellite Analysis with MSH2 and/or MLH1 Immunohistochemical Finding. Mismatch Repair Defective Tumours are Those Carcinomas Exhibited Loss of Either MLH1 or MSH2 Expressions by Immunohistochemistry

IHC	MSI		Total
	MSI negative	MSI positive	
MLH1 and MSH2 present	50	4	54
MLH1 loss	0	3	3
MSH2 loss	0	3	3

**Table 2:** Clinicopathological Characteristics for MSI Positive Cases

Case no.	Type*	Age	Ethnic	Stage	Microsatellite markers					IHC	
					BAT25	BAT26	D2S123	D5S346	D17S250	MSH2	MLH1
2	Intestinal	67	Chinese	-**	-	+	-	-	+	-	+
15	Intestinal	83	Chinese	-**	+	+	-	+	-	+	+
21	Diffuse	65	Chinese	Advanced stage	-	+	-	-	+	-	+
27	Intestinal	73	Chinese	Stage IV (T4N2M1)	-	+	-	+	+	+	-
28	Intestinal	63	Chinese	Stage IIIA (T3-4N2M0)	+	+	-	+	-	+	-
30	Intestinal	69	Chinese	Stage IB (T2N0M0)	+	+	-	-	+	+	-
35	Intestinal	55	Chinese	Stage IIB (T3N1Mx)	-	+	+	-	+	-	+
37	Intestinal	79	Malay	Stage III	-	-	+	-	+	+	+
41	Intestinal	54	Malay	Stage IIB (T3N1M0)	+	+	-	-	-	+	+
46	Intestinal	64	Chinese	Stage IIIA (T4N1M0)	+	+	-	-	-	+	+

*, Type of gastric cancer by Laurèn Classification; **, Patient’s file has been discarded

**Table 3:** Correlation of MSI with Clinicopathological Features

Variable	n	MSI negative	MSI positive	P*
		(n = 50)	(n = 10 )	
Age				0.183
< 50 years	11	11	0	
≥ 50 years	49	39	10	
Sex				1
Male	38	32	6	
Female	22	18	4	
Type (Laurén)				0.146
Intestinal	39	30	9	
Diffuse	21	20	1	
Lymph node spread				1
Absent	16	13	3	
Present	44	37	7	
Race				0.651**
Malay	14	12	2	
Chinese	44	36	8	
Indian	2	2	0	
Sites				0.334***
Distal	50	40	10	
Proximal	9	9	0	
Anastomotic	1	1	0	
*H. pylori *				0.675
Absent	47	40	7	
Present	13	10	3	

*, P values correspond to two-sided Fisher’s exact test; **, Analysis involving Chinese and non-Chinese; ***, Analysis involving tumour located at distal and proximal sites.

**Table 4:** List of MSI Positive MLH1 Mutation in Gastric Cancer. *Immunohistochemistry Status for MLH1

Case	Intron/exon	DNA sequence changes	Amino acid changes	Conserved domain region	Mutation type	Pathogenicity	IHC*
15	Intron 3	c.208-47delA		None	Deletion		Positive
27							Negative
28							Negative
41							Positive
15	Intron 8	c.677+72T>G		None	Insertion		Positive
27							Negative
28							Negative
46							Positive
27	Exon 9	c.757G>A	p.Val253Met	None	Missense	Neutral/NN:0.2409	Negative
15	Exon 12A	c.1048delC		None	Frameshift		Positive
27							Negative
28							Negative
30							Negative
27		c.1056T>C	p.Leu352Leu	None	Silent		Negative
28		c.1116T>C	p.Ser372Ser	None	Silent		Negative
15	Exon 12B	c.1227G>A	p.Gln409Gln	None	Silent		Positive
	Intron 12B	c.1409+1G>A		None	Insertion		Positive
30	Exon 13	c.1464G>A	p.Lys488Lys	None	Silent		Negative
15	Intron 16	c.1732-43G>C		None	Insertion		Positive
37		c.1896+49C>G		*Racemase-4 Superfamily*	Silent		Positive
30	Exon 16	c.1874A>C	p.Tyr625Ser	None	Missense	Pathogenic/NN: 0.6626	Negative
27	Exon 19	c.2118C>T	p.Gly706Gly	None	Silent		Negative

**Table 5:** List of MSI Positive *MSH2* Mutation in Gastric cancer. *Immunohistochemistry Status for *MSH2*

Case	Intron/exon	DNA sequence changes	Amino acid changes	Conserved domain region	Mutation type	Pathogenicity	IHC*
21	Exon 2	c.214G>A	Ala72Thr	None	Missense	Neutral/NN: 0.1368	Negative
46		c.326A>C	Asn109Thr	*MutS-I* Superfamily	Missense	Neutral/NN: 0.1445	Positive
2	Intron 9	c.1510+31insAA		None	Insertion		Negative
		c.1510+32insA					
15				None			Positive
35				*MutS-IV* Superfamily			Negative
41				None			Positive
46				None			Positive
21		c.1510+29insGT		None	Insertion		Negative
		c.1510+30insA					
2	Intron 13	c.2210+17G>C		None	Insertion		Negative
15	Exon 13	c.2018G>A	Glyn673Glu	*P-loop_NTPase* Superfamily	Missense	Pathogenic/NN: 0.7651	Positive
15	Exon 14	c.2260A>G	Thr754Ala	*P-loop_NTPase* Superfamily	Missense	Neutral/NN: 0.1280	Positive
41	Intron 14	c.2209-26G>T		None	Insertion		Positive
2	Intron 16	c.2635-25A>C		None	Insertion		Negative
35							Negative

**Figure 1:**
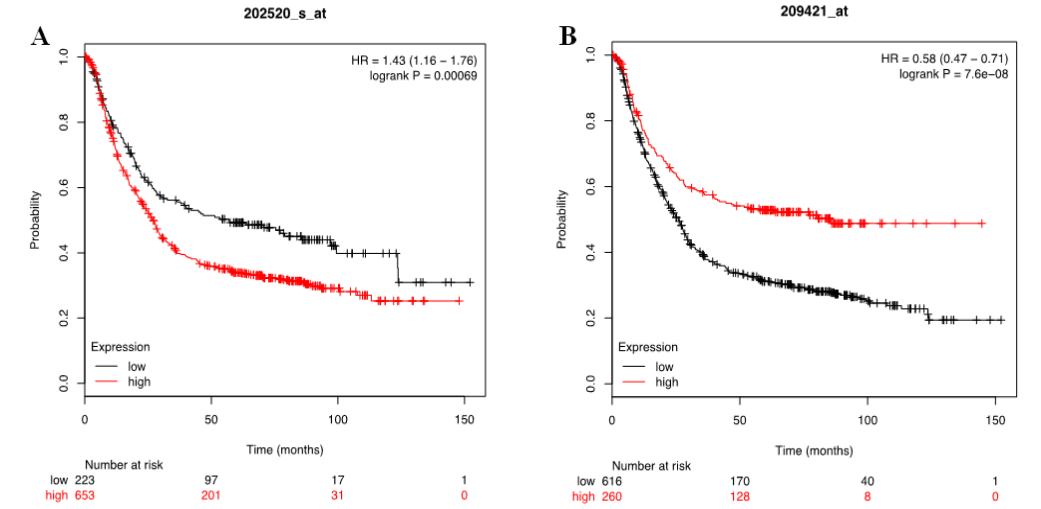
Disease Free Survival of Gastric Cancer Patients in Association with Transcriptomic MLH1 and MSH2 Expression Levels. (A) High MLH1 expression is significantly associated with worst overall survival in gastric cancer patients (HR = 1.43; p-value = 0.00069). Inverse correlation observed in high MSH2 gene expression displaying good overall survival in gastric cancer patients (HR = 0.58; p-value = 7.6e-08)

**Figure 2:**
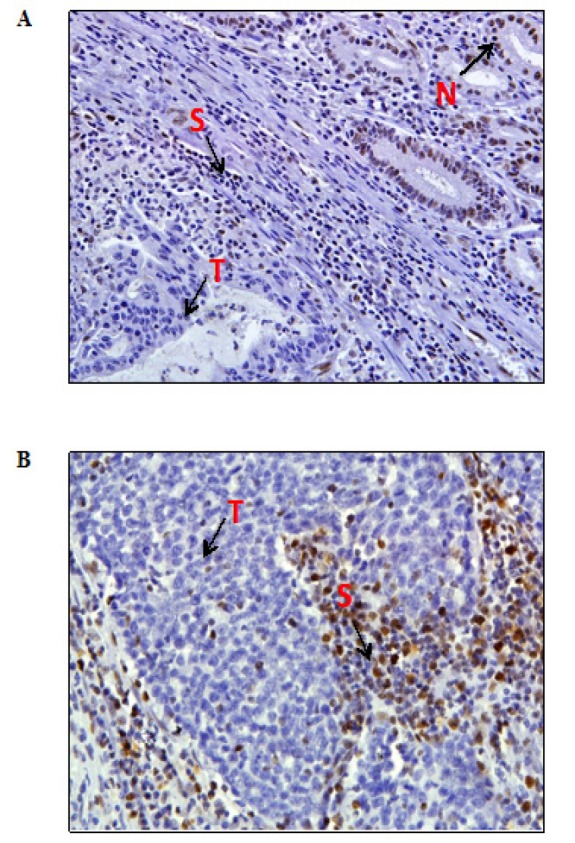
(A),Well differentiated intestinal type gastric cancer with positive MSI showing total loss of MLH1 protein expression in tumour cells (T). Normal gastric glands (N) as internal control showing positive staining; (B), Diffuse type gastric cancer with positive MSI showing negative staining for MSH2 protein in tumour cells (T). Stromal cells (S) with positive staining as internal control. (x 200 magnification)

**Figure 3:**
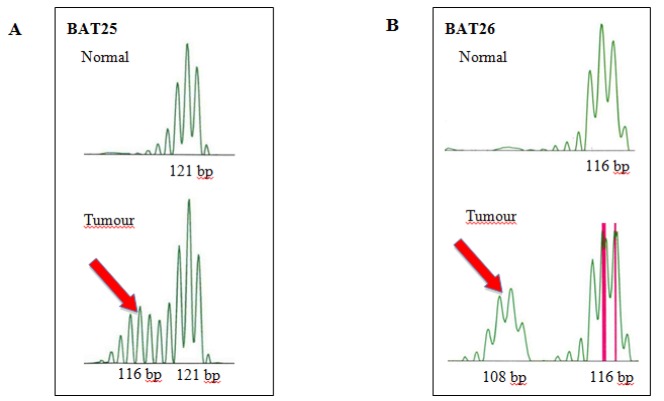
Electropherograph from a Case of Gastric Cancer with MSI Showing Additional Peaks in Tumour Compared to Matched Normal Tissue in Two Markers, (A) BAT25 and (B) BAT26

**Figure 4:**
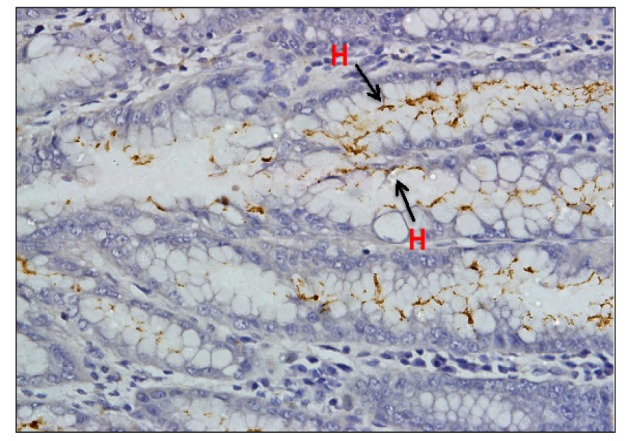
Presence of *H. pylori* Indicated by Brownish-Stained Organisms Located at the Luminal Surface of Gastric Mucosa Cells (Magnification x 400)

*Mutations in the MLH1 and MSH2 genes are negatively correlated with protein expression*

DNA sequencing analyses presented 13 and 9 mutations, in both MLH1 and MSH2 respectively. Amongst the detected mutations, only one mutation was previously reported flanking the 12B intronic region of the MLH1 (Case 15) while others were uniquely observed in this current study and never been reported in any other Lynch Syndrome studies as denoted in the MMR Genes Variance Database, International Society for Gastrointestinal Hereditary Tumours Database and Human Gene Mutation Database suggesting that these mutations are novel mutations. Our sequencing data noted mutation of G to A nucleotide substitution at region c.1409+1. This mutation was also reported in a single nucleotide polymorphism database (http://www.ncbi.nlm.nih.gov/sites/varvu) except for the mutation of Gly706Gly at exon 19 c.2118 of the MLH1 gene (Case 27). This silent mutation displayed changes of the single variant involving changes of C to T nucleotide. Altogether, the 5 novel mutations were located within the conserved domain regions of the Racemase-4 Superfamily and P-loop_NTPase superfamily (Table 4). Our sequencing data indicated all missense mutations detected were not pathogenic based on Pmut software (http://mmb.pcb.ub.es:8080/Pmut) indicating the mutations were not deleterious. A single missense mutation case is pathogenic involving exon 16 with negative expression of MLH1 protein. 

Interestingly, our data exhibited a novel frameshift mutation noting deletion of a cytosine at exonic region c.1048 (c.1048delC) flanking exonic region 12A of the MLH1 gene. This mutation was positively correlated with MSI positivity, overexpression of MLH1 protein and was positive with H.pylori UreA gene marker (data not shown). However, clinicopathological data was insufficient to associate these variables that could link this mutation to the disease.

The MSH2 also displayed novel mutations harbouring at highly conserved region MutS-IV superfamily with negative expression of MSH2 protein (Table 5). Inverse correlation was observed denoting the c.326A>C missense mutation (Case 46) also located in the MutS-IV conserved region with positive expressions of MSH2. However, this mutation is neutral suggesting it was non-pathogenic. One positively stained MSH2 protein displayed positive correlation with c.2018A>G missense mutation flanking the P-loop_NTPase superfamily conserved region and was pathogenic. 

## Discussion

A variety of techniques have been used for identification of tumours with mismatch repair (MMR) deficiencies ranging from PCR-based to immunohistochemistry (Fidalgo et al., 2000; Samimi et al., 2000). In this study, we combined these techniques to test its specificity to the determination of markers as well as whether the protein expression of the MLH1 gene and MSH2 are highly associated with mutations or whether the MSI may be contributed by mutations of the MLH1 and MSH2 genes. Immunohistochemistry was a complementary method for PCR-based where analysis for the DNA MMR proteins such as MLH1, MSH2, MSH6 and PMS2 are readily available on a clinical basis. IHC also provides information on the specific defective MMR gene involved and may be cost effective by limiting the number of genes to be sequenced to identify individual at risk. PCR-based method may detect cases that have abnormalities in any MMR genes (except certain cases of MSH6 gene defect) including those that are not covered by the IHC antibody panel (Frayling et al., 2005; Shia, 2008).

In this study, sensitivity of IHC testing against microsatellite positive results was slightly low due to four additional MSI positive cases showed intact MLH1 and MSH2 protein expressions. A similar false positive result by immunohistochemistry has been previously reported by other studies which utilised two antibodies in the assessment of MSI analysis (Cai et al., 2004; Modica et al., 2007). This may be due to low sensitivities of IHC staining of both MLH1 and MSH2 antibodies than MSI testing in determining MMR gene defect. However, by taking into account PMS2 and MSH6 staining could significantly increased the sensitivity of IHC resulting an equivalent value to that of MSI testing (Jong et al., 2004; Hampel et al., 2005; Southey et al., 2005). This may be due to heterodimers formation of MMR proteins that in their functional state MSH2 dimerizes with MSH6 to form functional complex MutS-α, while MLH1 dimerizes with PMS2 to form MutL-α. MSH2 and MLH1 proteins were the obligatory partners of their respective heterodimers. Their abnormalities can result in proteolytic degradation of their dimer and consequent loss of both the obligatory and secondary partner proteins. However, if the functions of MSH6 and PMS2 were affected, it does not contribute to concurrent loss of MSH2 and MLH1 protein as the function of the secondary proteins may be compensated by other proteins, such as MSH3, MLH3 and PMS1 (Shia, 2008). In addition, gene abnormalities in MLH1 and MSH2 may cause pathogenic mutations contributed to retained protein expression. This is due to the mutation itself that did not promote protein degradation or truncation, but missense mutation exhibited no difference with wild type polypeptides. Moreover, false positive staining for MLH1 can occur with protein truncating mutations and large in frame deletions in MLH1, where the mechanism is still unclear (Shia, 2008). The inclusion of the two antibodies had the capabilities to detect most abnormalities in MLH1 and MSH2 genes in addition to the mutation detection in MSH6 and PMS2 genes. 

Our study showed low frequency of MSI positive GCs closely related to previous studies using the five NCI panels of markers (Fang et al., 2003; Zaky et al., 2008). The clinicopathologic characteristics features associated with MSI positive tumours were advanced age of patients, distally located tumours and better overall prognosis (Hayden et al., 1997; Oliveira et al., 1998; Wu et al., 1998). However, insignificant results were obtained between MSI positive and negative group in this study. But, MSI positive tumours were more frequently located at the distal of the stomach and most of them were intestinal type by Laurén classifications and these observations are in line with previous study displayed MSI-H groups that represented loss of MMR proteins and associated with intestinal type. This also indicated its association with overall good prognosis (Karpinska-Kaczmarczyk et al., 2016). However, these data warrant a larger number of GCs samples for further evaluation to elucidate distinct prognosis between MSI tumours and clinicopathological characteristics.

*H. pylori* status have been used to classify GC subtypes and also indicates the risk and prognostic factors of gastric carcinoma. In this study, *H. pylori* was determined by IHC staining. The prevalence of *H. pylori* infection by IHC staining was higher in Chinese (12/13, 92.3%) compared to Malay (1/13, 7%). This result concurred the outcome of GCs itself which is high in Chinese compared to the Malay and Indian in this study as well as the incidence reported in cancer registries from Malaysia (Zainal and Nor Saleha, 2011) and Singapore (Curado et al., 2007). 

Previous studies have shown a significant association between GCs with MSI positive and *H. pylori* infection (Wu et al., 1998; Leung et al., 2000; Li et al., 2005) but some discovered inverse correlation between the two indicators of GCs (Lin et al., 1995; Kashiwagi et al., 2000; Rugge et al., 2005). In this study, three MSI positive were found in 13 patients with *H. pylori* infection without positive correlation between them. Overall, the association between the *H. pylori* infection and MSI positive remain unresolved among researchers (Kashiwagi et al., 2000). A recent review suggesting that *H. pylori* infection act as a synergistic factor in GCs but not a direct factor causing carcinogenesis by altering the gene expression (Shokal and Sharma, 2012). It has been shown that H.pylori infection was infrequently detected in a larger cohort of GCs, which successfully classified four distinct GCs subtypes. This data by TCGA proposed that GC was subdivided into those of which had Epstein-Barr virus infection with lacking hypermethylation status of the MLH1 gene (Matsusaka et al., 2011; Wong et al., 2015). Another subtype was denoted as those having MSI resulted in hypermutated tumours and high hypermethylation status at the MLH1 promoter regions. Genome instability and chromosomal instability were also denoted as GC subtypes affecting the diffuse and intestinal GCs (Wong et al., 2015). Inverse outcomes were seen in another study displayed no correlation of MSI status in each of the classified subtypes (Cristescu et al., 2015). Hence, the urgency in determining the right molecular markers and driver mechanisms in GCs is crucial for improvement of the GC management.

BAT26 is a mononucleotide marker harbours specificity and sensitivity in identifying MSI positive GC (Halling et al., 1999; Guo et al., 2001; Falchetti et al., 2008). Nine over ten GCs showed MSI positive cases by this marker. This might be due to the quasimonomorphic profile (shift of allele is greater than 0 but less than 1 base pair) of BAT26 that amplifies at higher efficiency than the larger normal allele (Brennetot et al., 2005). However, BAT26 should not be solely depended on (Halling et al., 1999). First, it was inadequate to only assess the level of MSI (Bacani et al., 2005; Zaky et al., 2008) and displayed variable electrophoretic patterns in certain cases. Second, it will overestimate the MSI positive phenotype (Bacani et al., 2005). Apart from that, sensitive markers identifying MSI were reported to have different results in different populations (Buhard et al., 2006). Therefore, markers selection should be carefully made when analyses are performed in people with different geographic and genetic backgrounds (Sepulveda et al., 1999). 

Studies lately have shown that there is an equivalent sensitivity between IHC and MSI analysis in determining MMR gene abnormalities (Jong et al., 2004; Hampel et al., 2005; Southey et al., 2005). In this study, however determining affected *MMR* genes by IHC using MLH1 and MSH2 antibodies may lead to false negative MSI. It has been shown that PCR-based MSI detection was relatively low in GC, however higher prevalence of MLH1 and PMS2 were observed (Karpinska-Kaczmarczyk et al., 2016; Mathiak et al., 2017). Therefore, four antibodies panel (MLH1, MSH2, PMS2 and MSH6) are compulsory panel markers for further evaluation in order to increase sensitivity of IHC as predictive value that is almost equivalent to that of MSI analysis. 

Mutations detected in this study was positively correlated with MSI positive but displayed indecisive patterns of *MLH1* and *MSH2* expression levels even when the mutations fall within the exonic regions with high pathogenic effect (Table 4 and 5). This scenario may be associated with hypermethylation of the *MLH1* and *MSH2 *promoter region that represses the expressions of these genes as seen in endometrial cancer with HNPCC syndrome (Chadwick et al., 2001). It was also clear that high MLH1 and MSH2 expressions represented worst survival in GC suggesting high intratumoural heterogeneity within the tumour samples that contributed in GC treatment resistance. Nevertheless, other mutations were rather novel in this study, hence, further assessments of its functional roles is fully recommended to further explicate its prognostic values in GCs. As previously mentioned, MSI status varies greatly in different population and ethnicity. It would be of a great value then to have higher number of samples to assess the different groups of GCs (intestinal, diffuse and mixed) and the stages to observe strong correlations of the clinical data and genomic instability to rule out surrogate markers to rule out prognosis for novel therapeutic intervention in GCs. 

 In conclusion, the prevalence of MSI in GC was 16.7% involving mainly intestinal type cancers with all distally located and this compares well with published results. Based on recent work of MMR gene abnormalities detection, four antibodies panel (MLH1, MSH2, PMS2 and MSH6) should be utilised instead of two (MLH1 and MSH2) in order to increase sensitivity of IHC result as a potential predictive value. This detection method has equivalent to that of MSI analysis. Having said that, larger cohorts are required to have a statistically significant profiles of GCs in Malaysia.

## Funding

This work was supported by an Intensification Research Priority Area (IRPA) Grant (No. 06-02-02-0057-PR-0073/05-04) from the Ministry of Science and Technology, Malaysia.

## Conflict of interest statement

No conflicts of interest were disclosed throughout the study.
